# Correlation between religious coping, demographic and fertility factors, and pregnancy anxiety of Iranian primiparous women: a cross-sectional study

**DOI:** 10.1186/s12888-022-03922-2

**Published:** 2022-04-28

**Authors:** Foruzan Mirzaee, Seyedeh Batool Hasanpoor-Azghady, Leila Amiri-Farahani

**Affiliations:** Department of Midwifery and Reproductive, Nursing Care Research Center (NCRC), School of Nursing and Midwifery, Iran University of Medical Sciences, Tehran, Iran

**Keywords:** Pregnancy, Anxiety, Religious coping, Demographic and fertility variables

## Abstract

**Background:**

Anxiety during pregnancy can have side effects for both the mother and the baby. Therefore, it is necessary to study the factors that affect anxiety during pregnancy. This study aimed to investigate the role of religious coping and demographic and fertility factors in predicting pregnancy anxiety in Iranian primiparous women.

**Methods:**

We conducted a cross-sectional study on 300 primiparous women (*n* = 100 in each trimester of pregnancy) referred to seven health centers affiliated to the Iran University of Medical Sciences, Tehran, Iran. The sampling method was multistage. It lasted from July 2018 till August 2019. Data collection tools included the demographic and fertility questionnaire, valid and reliable Iranian Religious Coping Scale (IRCS), and standard State-Trait Anxiety Inventory (STAI).

**Results:**

Religious practices, benevolent reappraisal, and active religious coping had a significant inverse relationship with state and trait anxiety. Whereas negative and passive religious coping had a significant direct relationship with state and trait anxiety. The mean scores of state anxiety had a significant relationship with the women’s education, spouse's education and occupation, economic status and housing status. There was no relationship between state anxiety and fertility variables. Based on multiple linear regression, negative and active religious coping predicted 27% of state anxiety and 15% of trait anxiety. Among these two variables, the negative religious coping was the more effective in predicting state and trait anxiety.

**Conclusion:**

With increasing positive religious coping, the anxiety of pregnant women decreased. Whereas with increasing negative religious coping, their anxiety increased. Our results emphasize the role of negative religious coping in predicting pregnancy anxiety.

## Background

Pregnancy can be associated with stress due to physiological and psychological changes in women [1]. Especially in nulliparous women, where pregnancy is a new experience, it can lead to anxiety if stress is not managed [2]. Pregnancy-related anxiety may be a risk factor for postpartum depression and negative consequences for the child [3].

Dennis et al. reported the prevalence of anxiety during the perinatal period at 13.6 to 22.8% [4]. Madhavanprabhakaran et al. showed the general anxiety moderate (48.6%) to severe (48.4%) during the first trimester and moderate (71%) to severe (29%) during the third trimester [5]. Studies have shown that the mean anxiety in the third trimester of pregnancy is higher compared to the first and second trimesters [5, 6]. Some studies have reported that anxiety level is high in the first trimester of pregnancy, low in the second trimester, and increase in the third trimester [7, 8]. The prevalence of state and trait anxiety in pregnant women in Iran is 42.6% and 45.3%, respectively [9].

During pregnancy, mothers focus their thoughts on fetal health, fear of miscarriage, physical and mental abnormalities of the baby. It causes anxiety in them [5] that can have side effects for the child and mother in the following years. Consequences in the child include nutritional problems, increased rates of asthma, and respiratory infections [10], poor physical development, mood disorders and future medical problems of the child [11], neonatal colic, behavioral disorders during puberty and abnormal social behaviors [12]. Complications in the mother include muscle pain, sleep disturbance [13], poor quality of life in all aspects of life, especially sexually [14, 15], delayed response to their baby's crying, and an incomplete emotional relationship with their babies [16]. Given these consequences, pregnant mothers need coping to overcome pregnancy anxiety [17].

Coping is a specific behavioral and psychological effort that individuals use to control, tolerate, reduce, or minimize stressful events [18]. Pargament et al. believe that religion is part of the coping process because it provides resources for individuals to evaluate situations differently and increases their ability to cope with the status [19]. Religious coping reflects an individual's efforts to use religious teachings to understand coping with life's stressful events and experiences [20]. Religious coping, however, has a positive implicit meaning but can be both positive and negative. Positive religious coping indicates a secure relationship with the transcendent force, a sense of connection with spirituality, others, and a benevolent view of the world [21]. Positive religious coping with challenging natural science explanations, semantics and redefining stressors as an opportunity for spiritual growth is an impact factor [22]. Applying a positive religious coping by connecting with the surrounding environment and increasing the meaning of life reduces negative emotions and bring happiness [4]. Negative religious coping is a less secure relationship with God and a weaker perspective of the world [21], including a negative evaluation of God as a punisher, anger and doubt about the power of God [22]. Guardino and Dunkel Schetter reported that positive religious coping is inversely related to stress levels in pregnant women [23]. Puente et al. showed that 17.1% of pregnant women use religious coping during pregnancy [24]. The results of research in a genetic counseling center showed that 92.8% of pregnant women who referred to this center used religious coping at least one or more times [25].

During pregnancy, positive religious coping includes praying, replacing negative dreams with positive feelings and having positive dreams, giving thanks in church, singing, and emotional support [26]. In Islamic countries, pregnant women use religious coping such as listening to the Quran, praying, relying on God, repeating religious remembrances, patience, and spiritual and religious psychotherapy [27]. The results of a qualitative study showed that experiencing the first pregnancy is associated with three themes of spiritual awareness (presence of divine truth in mothers' lives), the origin of life (mother as the giver of life) and divine reality (God supports the life of pregnant mothers), [28]. The study of Jabbari et al. showed that listening to the Quran reduced state and trait anxiety of pregnancy compared to the control group [29]. Although previous studies have often shown a positive effect of religious coping on anxiety, some studies have not found a significant relationship between religion/spirituality and pregnancy anxiety [30–32]. Even Mann et al. showed Religiosity/spirituality was significantly associated with an increase in negative stress experiences in pregnant and postpartum women who chose English language instruments. But there was no relationship between Religiosity/spirituality pregnant and postpartum women who chose Spanish language instruments and negative stress experiences [33]. In this regard, Peñacoba‐Puente et al. reported that among demographic factors, fertility and coping strategies, one of which was religious coping, only age was the predictor of both variables anxiety and depression in Spanish Pregnant Women [31]. The results of Peter et al.'s study on100 Pakistani pregnant women also found no significant relationship between religious coping of pregnant women and anxiety [30]. Contradictory study results can have different causes. Perhaps one of them is the lack of a designed or psychoanalyzed religious coping questionnaire in the context of the culture of the study community, which has forced researchers to use religious coping questionnaires from other communities. Because of the interaction between religion and culture, we used the Iranian Religious Coping Scale (IRCS) to measure religious coping in the present study.

Regarding demographic and fertility factors, some studies have briefly noted the association between demographic variables and fertility and anxiety. A study found that pregnancy anxiety was higher in women with unwanted and nulliparous pregnancies [34]. Gurung et al. reported that Younger women and women with higher education were more anxious during pregnancy. But high-income pregnant women had lower anxiety than their counterparts [35].

Since one of the goals of midwifery is to manage pregnancy anxiety, Because it has adverse effects on mother and child, this study was conducted to investigate the role of religious coping and demographic and fertility factors in pregnancy anxiety of Iranian primiparous women for several reasons. The contradictions in the results of studies, the special place of religion in Iranian society, and the importance of examining the role of demographic and fertility variables in predicting pregnancy anxiety, perhaps the results of this study can provide a more comprehensive view for experts who provide care for pregnant women.

## Methods

This cross-sectional study was conducted on 300 primiparas women (*n* = 100 in each trimester of pregnancy) who had been selected from seven health centers affiliated with the Iran University of Medical Sciences. This university covers seven districts in Tehran, the capital of Iran. The sampling method was multistage. First, we selected one health center from each region at random, then the eligible individuals were determined in each center by quota method. Continuous sampling was conducted at each center between July 2018 till August 2019. We used G * power software to calculate the required sample size. The sample size with the confidence level of 95%, test power of 9.0 and medium effect size based on Cohen classification was determined to be 272 individuals considering 19 predicting variables. We enrolled 300 people. The inclusion criteria were; having the ability to read and write, being in the first marriage, living with a spouse, having a singleton pregnancy, and being biological parents of the fetus. Exclusion criteria were; occurrence of stressful life events in the last six months, using drugs or any medications related to mental disorders, having a chronic systemic disease, high-risk pregnancy, abnormality and pathological problems in the fetus based on ultrasound examination.

Data collection tools included the demographic and fertility questionnaire, the Iranian Religious Coping Scale (IRCS), and State-Trait Anxiety Inventory (STAI). Aflakseir and Coleman designed the IRCS. This scale consists of 22 items. Its scoring system is based on a 5-point Likert scale ranging from 0 (not at all) to 4 (a great deal). It also has five subscales including, the practice of religious coping (6 items), benevolent reappraisal (6 items), negative religious coping (4 items), and passive and active religious coping (3 items each). This questionnaire does not have a total score. High scores on each subscale indicate high religious coping on that subscale. Cronbach’s alpha was 0.89 for practice religious coping, 0.79 for benevolent reappraisal, 0.79 for negative religious coping, and 0.72 and 0.79 for passive and active religious coping, respectively [36]. Aflakseir and Coleman implicitly stated that among the five subscales of IRCS, active religious coping, benevolent evaluation, and practices of religion are positive religious coping, and negative and passive religious coping are negative religious coping [36].

The STAI is a self-reporting inventory. It has two subscales that each composed of 20 items. The state anxiety subscale is a measure of how the participant is feeling “at this moment”. The trait anxiety subscale is a measure of how the participant “generally feels”. Response options range from one (not at all) to four (very much so) for both the state and trait subscales. Higher scores indicate higher anxiety, with total scores on each subscale ranging from 20 (not anxious) to 80 (very anxious) [37]. The STAI has been widely validated in the general population [38] and is one of the most common measures used in research to assess the anxiety of pregnant women [39].

Sampling began after approval of the project by the ethics committee of Iran University of Medical Sciences with the code (IR.IUMS.REC.1397.545). After explaining the objectives of the study and the principle of confidentiality, the researcher obtained informed written consent from eligible subjects and then asked them to complete the demographic and fertility, IRCS, and STAI questionnaires by self-administered.

Data were analyzed by SPSS software version 22 using independent t-test, one-way ANOVA, Kruskal-Wallis, Pearson correlation, and multiple linear regression. The multiple linear regression model was used to determine the simultaneous effect of the variables, each of which was related to the dependent variable using the Enter method. Before the implementation of multivariate analysis, we checked the regression assumptions, such as normality of the residuals, homoscedasticity, multicollinearity, and independence of the residuals. The significance level for all tests was *p* < 0.05.

## Results

The age range of women was between 18 and 42 years with a mean and standard deviation of 26.39 ± 5.32. The majority of them (77.3%) were housewives. We presented more information about the demographic and fertility characteristics of the samples in Table 1.

**Table 1:** Demographic and fertility characteristics in primiparous women (*n* = 300)

**Characteristics**	**N (%)**	**Characteristics**	**N (%)**
**Women's age (years)**		**Housing status**	
<21	64 (21.3)	owner	97 (32.3)
21-25	68 (22.7)	Rent	153 (51)
26-30	105 (35)	Living with the husband's family	49 (16.3)
>30	63 (21)	Living with a female family	1 (0.4)
**Women’s education**		**Abortion history**	
< High school	59 (19.7)	Yes	52 (17.3)
High school	100 (33.3)	No	248 (82.7)
Academic	141 (47)	**Whether or not the pregnancy was wanted from the women’s point of view?**	
Spouse's education		Wanted	269 (89.7)
< High school	66 (22)	Unwanted	31 (10.3)
High school	95 (31.7)	Whether or not the pregnancy was wanted from the spouse's point of view?	
Academic	139 (46.3)	Wanted	272 (90.7)
Women’s occupation		Unwanted	28 (9.3)
Housewife	233 (77.7)	Whether or not the sex of the fetus was wanted from the women's point of view?	
Employed	67 (22.3)	Wanted	161 (53.7)
Spouse's occupation		Unwanted	42 (14)
Employee	96 (32)	uncertain	
Free	147 (49)	Whether or not the sex of the fetus was wanted from the spouse's point of view?	
manual worker	38 (12.7)	Wanted	161 (53.7)
Unemployed	19 (6.3)	Unwanted	42 (14)
Economic status		uncertain	97 (32.3)
Favorable	184 (61.3)		
Relatively favorable	80 (26.7)		
Undesirable	36 (12)		

We calculated the mean of the religious coping subscales based on 0 to 100 to compare them (Table 2).

**Table 2:** Means and standard deviations subscales of religious coping and state-trait anxiety in primiparous women (*n*=300)

**Subscales of religious coping**	**Mean**	**SD**	**Mean based on one hundred**
Practice	14.75	5.5	61.45
Benevolent reappraisal	14.23	4.82	59.29
Negative	3.43	3.08	21.43
Active	8.55	2.65	71.25
Passive	3.36	2.23	28
State anxiety	37.49	9.50	
Trait anxiety	37.46	9.33	

The relationships between demographic and fertility characteristics and state anxiety are presented in Tables 3 and 4.

**Table 3:** The relationship between demographic characteristics and state anxiety in primiparous women (*n*=300)

Characteristics	State anxiety	
	Mean (SD)	*P*-value
Women’s age (years)		
<21	38.78 (9.60)	
21-25	37.44 (11.17)	^*^P= 0.47
26-30	36.93 (8.17)	
>30	31.17 (9.73)	
^**a**^Women’s education		
< High school	39.71 (10.53)	^**^P= 0.001
High school	38.80 (9.64)	
Academic	35.64 (8.63)	
^**b**^Spouse’s education		
< High school	39.68 (10.04)	^**^P= 0.01
High school	38.37 (9.84)	
Academic	35.86 (8.74)	
Woman’s occupation		
Housewife	38.01 (9.57)	^***^P= 0.07
Employed	35.69 (9.10)	
^**c**^Spouse’s occupation		
Employee	34.95 (8.24)	^*^P= 0.001
Free	37.67 (9.37	
Manual worker	40.08 (9.64)	
Unemployed	43.84 (26.12)	
^**d**^Economic status		
Favorable	35.20 (8.21)	^*^P= 0.001
Relatively favorable	41.51 (9.67)	
Undesirable	40.28 (11.63)	
^**e**^Housing status		
owner	35.87 (7.65)	^*^P= 0.01
Rent	37.31 (9.47)	
Living with the husband’s family	41.20 (11.79)	

^*^Kruskal–Wallis ^**^One way ANOVA ^***^Independent t-test

^****^The option of living with a female family with low frequency (1) was not considered in the analysis

^a^According to the Tukey test, the mean state anxiety score of pregnant women with academic degrees was lower than those with less than high school (*P* = 0.01) and high school (*P* = 0.02) education

^b^According to the Tukey test, the mean score of state anxiety of pregnant women who had husbands with academic education was lower than women whose husbands had less than high school education (*P* = 0.01)

^c^The pairwise comparisons showed that the mean state anxiety score of pregnant women whose husbands were employees was lower than women whose husbands were unemployed (*P* = 0.01) and manual workers (*P* = 0.02)

^d^The pairwise comparisons also showed that the mean score of state anxiety of pregnant women with favorable economic status was lower than women with relatively favorable (*P* = 0.02) and unfavorable (*P* = 0.001) economic status

^e^The pairwise comparisons revealed that the mean score of state anxiety of pregnant women living in a private home was lower than those living with their husbands’ families (*P* = 0.01)

**Table 4:** The relationship between fertility characteristics and state anxiety in primiparous women (*n*=300)

Characteristics	State anxiety	
	Mean (SD)	*P*-value
Pregnancy trimester		
First trimester	36.57 (9.00)	^*^P= 49
Second trimester	36.98 (9.01)	
Third trimester	41.45 (10.00)	
Abortion history		
Yes	37.31 (9.83)	^**^P= 0.87
No	37.53 (9.45)	
Whether or not the pregnancy was wanted from the women’s point of view?		
Wanted	37.30 (9.14)	^**^P= 0.41
Unwanted	39.19 (12.29)	
Whether or not the sex of the fetus was wanted from the women’s point of view?		
Wanted	37.61 (9.06)	^***^P= 0.43
Unwanted	39.14 (11.56)	
Whether or not the pregnancy was wanted from the spouse’s point of view?		
Wanted	37.46 (9.28)	^**^P= 0.83
Unwanted	37.86 (11.63)	
Whether or not the sex of the fetus was wanted from the spouse’s point of view?		
Wanted	37.56 (9.21)	^*^P= 0.28
Unwanted	39.36 (11.05)	
uncertain	36.58 (9.24)	

One way ANOVA **Independent t-test ***Kruskal–Wallis^*^

The relationship between subscales of religious coping and state-trait anxiety are presented in Table 5.

**Table 5:** The relationships between religious coping and state-trait anxiety in primiparous women

Subscales of religious coping	State anxiety		Trait anxiety	
	r	*P*- value	r	*P*- value
Practice	-0.16	0.005	-0.19	0.001
Benevolent reappraisal	-0.26	0.001	-0.23	0.001
Negative	0.40	0.001	0.37	0.001
Active	-0.24	0.001	-0.20	0.001
Passive	0.35	0.001	0.35	0.001

The variables that had a significant relationship with state anxiety entered in the multiple linear regression model with Enter method. Negative and active religious coping remained in the model. According to the results, the mean score of state anxiety was higher in pregnant women who use more negative religious coping (B=.79, 95% CI=.42 to1.16, *p*<.001) and lower in pregnant women who use more active religious coping (B=-.49, 95% CI= -.93 to -.05, *p*=.029). Thus, according to the results, 27% of state anxiety was predicted by negative and active religious coping. Among these two variables, the negative religious coping was the more effective in predicting state anxiety (Table 6).

**Table 6:** Results of multiple linear regression analysis to investigate the effect of religious coping and demographic characteristics on state anxiety in primiparous women (*n*=300)

Independent variable		B Coefficient	Standardized Coefficient	Statistics	*P*-value	95% CI (L / H)	*R*^2^
Practice		.27	.15	1.97	.050	.001/.54	.27
Benevolent reappraisal		-.32	-.16	-1.93	.053	-.65/.005	
Negative		.79	.25	4.19	< 0.001	.42/1.16	
Active		-.63	-.17	-2.79	.006	-1.08/-.188	
Passive		.15	.035	.61	.537	-.32/.63	
Women’s education	<High school	1.28	.54	.73	.462	-2.15/4.72	
	High school	.49	.02	.39	.691	-1.95/2.95	
	Academic	Reference Category					
Spouse’s education	<High school	.26	.01	.14	.882	-3.29/3.82	
	High school	.75	.03	.58	.561	-1.78/3.28	
	Academic	Reference Category					
Spouse’s occupation	Employee	-1.95	-.09	-.73	.464	-7.19/3.29	
	Free	-1.93	-.10	-.81	.416	-6.60/2.73	
	Manual worker	-.73	-.02	-.28	.773	-5.76/4.28	
	Unemployed	Reference Category					
Economic status	Favorable	-2.06	-.10	-1.09	.274	-5.77/1.64	
	Relatively favorable	2.52	.11	1.35	.175	-1.13/6.17	
	Undesirable	Reference Category					
Housing status	owner	-2.58	-.12	-1.61	.107	-5.73/.56	
	Rent	-2.69	-.14	-1.87	.062	-5.53/.13	
	Living with the husband’s family	Reference Category					

All subscales of religious coping that had a significant relationship with trait anxiety entered in the multiple linear regression model with Enter method. Negative and active religious coping remained in the model. According to the results, the mean score of trait anxiety was higher in pregnant women who use more negative religious coping (B=.91, 95% CI=.54 to1.29, *p*<.001) and lower in pregnant women who use more active religious coping (B=-.47, 95% CI= -.98 to -.01, *p*=.041). Thus, according to the results, 15% of trait anxiety was predicted by negative and active religious coping. Among these two variables, the negative religious coping was the more effective in predicting trait anxiety (Table 7).

**Table 7:** Results of multiple linear regression analysis to investigate the effect of religious coping on trait anxiety in primiparous women (*n*=300)

Independent variable	B Coefficient	Standardized Coefficient	Statistics	*P*-value	95% CI (L/ H)	*R*2
Practice	.01	.009	.10	.918	-.26/.29	.15
Benevolent reappraisal	-.03	-.01	-.20	.834	-.37/.30	
Negative	.91	.30	4.79	< 0.001	.54/1.29	
Active	-.47	-.13	-2.05	.041	-.93/-.01	
Passive	.23	.05	.92	.356	-.26/.72	

## Discussion

The present study aimed to investigate the role of religious coping and demographic and fertility factors in predicting pregnancy anxiety in Iranian primiparous women. Findings showed religious practices, benevolent reappraisal, and active religious coping had a significant inverse relationship with state and trait anxiety. Also, the subscales of negative and passive religious coping had a significant direct relationship with state and trait anxiety. In a study conducted by Lucero et al. on primiparous pregnant women, the results showed that negative religious coping in pregnant women was associated with increased anxiety and decreased pregnancy satisfaction [40], which is consistent with the findings of the present study. The results of our study are also similar to the results of the study by Hamilton et al. that showed that positive religious coping is significantly associated with mid-pregnancy and late-pregnancy anxiety, and with increasing positive spiritual coping, pregnant women's anxiety level decreases [41]. Another study by Mann et al. on 404 low-risk pregnant women found a significant relationship between spiritual experiences, religious practices and pregnancy anxiety [42]. Among the studies conducted in Iran, we can refer to the research of Dilgony et al. on 450 pregnant women to determine the relationship between spiritual health and anxiety during pregnancy. The results showed a significant inverse relationship between pregnancy anxiety and spiritual health [43]. In line with the findings of our study, in the study of Foruzandeh Hafshejani et al., there was also a significant inverse relationship between pregnancy anxiety and spiritual well-being [44]. The results of another study showed that there is a significant negative relationship between the Islamic lifestyle and anxiety of 300 Iranian pregnant women [45].

Contrary to the findings of our study, the study of Peñacoba‐Puente et al. on122 low-risk pregnant women in the first and third trimesters, who were attending a public hospital in Madrid, showed that religious coping was not significantly associated with anxiety and depression during pregnancy [31]. Also, the results of the study of Peter et al. on 100 Pakistani pregnant women did not find a significant relationship between religious coping of pregnant women and anxiety [30]. Mann et al. also found no significant correlation between religion/spirituality and pregnancy stress in Hispanic women [33]. The disagreement in the results studies may be due to differences in sample size and tools, especially the tools and items related to religious coping, which vary depending on the religion and culture of each country.

Regarding demographic variables, the results of the present study showed that the mean state anxiety score of the samples with academic education was lower than the samples with less than high school and high school education. In participants whose spouses had an academic education, the mean score of state anxiety was lower than those whose spouses had a less than high school education. Consistent with our results, the findings of other studies show that increasing the level of education is associated with a decrease in anxiety [46–49]. But the results of two studies show that higher education is associated with more anxiety [35, 50]. The experience of the researchers in the present study in Iran shows that highly educated women are more likely to seek information through social media, so getting information support, especially during pregnancy and childbirth is a protective factor for them. In the present study, there was no relationship between state anxiety and women’s occupation. It is consistent with the results of the Boakye-Yiadom et al. [51]. However, Alqahtani reported that employed women had lower anxiety [52].

The findings of our study showed that the mean score of state anxiety of women with favorable economic status was lower than women with relatively favorable and unfavorable economic status. Regarding the status of housing, the mean scores of state anxiety of pregnant women who lived with their husbands' families were higher than those were in a private home. However, Alqahtani et al. found no relationship between anxiety, economic status and housing in pregnant women [52].

Regarding fertility variables, the results of our study showed that there was no relationship between state anxiety and variables of gestational age, history of abortion, wanted or unwanted pregnancy, and whether or not the sex of the fetus was wanted. In our study, although women in the third trimester of pregnancy had a higher mean anxiety score than pregnant women in the first and second trimesters, the difference was not statistically significant. In this regard, the results of several studies show no relationship between gestational age and pregnancy anxiety [51, 52]. But contrary to our results, the study of Kotimäki et al. on 2763 pregnant women found a significant difference between anxiety and gestational age. They showed anxiety in the second trimester was higher in pregnant women compared to other trimesters [53]. In another study conducted on 2705 pregnant women in Turkey, gestational age was significantly associated with anxiety, so that the younger the gestational age, the lower the level of anxiety was [54]. The results of a study by Gunning et al. on 215 English pregnant women showed that the mean score of state anxiety in the third trimester was higher from other trimesters [55]. Perhaps the reason for these discrepancies in the mentioned studies is the different sample sizes in different pregnancy trimmers and the cultural context of pregnant women.

To compare some fertility variables with the findings of other studies, we could not find a study with extensive searches. Some studies that examined the relationship between gestational anxiety and some variables had sideways reference to fertility variables. The results of these studies were inconsistent with the present study, such as the study of Barjesteh et al. that showed higher levels of anxiety were associated with older age [50]. In another study, anxiety increased with a history of abortion and unwanted pregnancy [52]. Perhaps the reason for different results is the variety of sample sizes of studies and the research community, which in our study were only primiparous women, and in other studies were primiparous and multiparous women.

### Research limitations

We had several limitations in our study, including:

We collected the data based on self-administered, the response to some items may have been influenced by individual characteristics and recognition. Variables such as personal characteristics, accuracy, honesty, and mental state of pregnant women in responding to the items were effective on the outcome of the research, which was beyond the control of the researchers.

We selected all samples from health centers, which may limit the generalization of results. However, health centers provide the main health services for pregnant women in Iran.

## Conclusion

There was a significant relationship between anxiety and religious coping in pregnant women. As the use of religious practices, religious benevolent evaluation, and active religious coping increased, the anxiety of pregnant women decreased, but on the other hand, as the negative and passive religious coping increased, the anxiety of pregnant women increased. State anxiety during pregnancy was associated with several variables, among which the negative religious coping had great impact on pregnancy anxiety. Paying attention to these variables can help develop appropriate counseling or educational programs for women's mental health during pregnancy.

## Data Availability

The datasets used and analyzed during the current study are available from the corresponding author on reasonable request.

## Abbreviations

IRCS: Iranian Religious Coping Scale
STAI: State-Trait Anxiety Inventory
